# Hydrocarbon pneumonitis following liquid paraffin aspiration during a fire-eating performance: a case report

**DOI:** 10.1186/1752-1947-2-214

**Published:** 2008-06-19

**Authors:** Efrosyni Mylonaki, Vasileios Voutsas, Dimitrios Antoniou, Despina Papakosta, Theodoros Kontakiotis, Anna Skordalaki, Evagelos Vafiadis, Pandora Christaki

**Affiliations:** 1 Second Pulmonary Clinic, G Papanikolaou General Hospital, Thessaloniki, Greece

## Abstract

**Introduction:**

Hydrocarbon pneumonitis is an acute, intense pneumonitis resulting from aspiration of volatile hydrocarbon compounds with low viscosity and surface tension, most of which are members of the paraffin, naphthene and aromatic classes.

**Case presentation:**

Six hours after participating in a party for teenagers, a 16-year-old boy developed dyspnea, cough, a fever (39°C) and chest pain. A chest radiograph showed infiltration in the right middle lobe. The patient reported alcohol abuse during the party and an episode of vomiting a few hours thereafter. He also reported practicing a fire-eating performance at the party using liquid paraffin, but was unaware of inhaling any of it. The radiographic infiltration was diagnosed as an aspiration pneumonia and he was treated at the local health center with antibiotics. Five days later, because of clinical deterioration, he was referred to a pulmonary clinic. A chest computed tomography scan was performed which showed consolidation with an air bronchogram in the right middle lobe and areas of atelectasis and ground glass opacities in the middle and lower right lobes. Spirometry revealed severe restriction of lung function. A bronchoscopy revealed inflamed, hyperemic mucosa. Bronchoalveolar lavage fluid revealed lipid-laden alveolar macrophages, which were detected by lipid staining, and neutrophilia. The patient was finally diagnosed with hydrocarbon pneumonitis and he was treated with systemic steroids and antibiotics. After 6 days of treatment there was complete clinical and significant radiologic regression.

**Conclusion:**

Hydrocarbon pneumonitis should be included in the differential diagnosis of pneumonias. Recent exposure to volatile hydrocarbons provides a basis for clinical diagnosis, as symptoms and radiologic findings are not specific.

## Introduction

Hydrocarbon pneumonitis is an acute, intense pneumonitis resulting from aspiration of volatile hydrocarbon compounds with low viscosity and surface tension, most of which are members of the paraffin, naphthene and aromatic classes [1]. Most cases are seen in children younger than 6 years of age, and result from unintentional aspiration of volatile hydrocarbon compounds. Most of the remaining cases are occupational exposures, such as fire-eaters and workers in the petrochemical industry. We report a case of hydrocarbon pneumonitis in a 16-year-old boy following liquid paraffin aspiration during a fire-eating performance.

## Case presentation

A 16-year-old boy was admitted to the local health center for evaluation of dyspnea, cough, chest pain and a body temperature of 39°C. He was normotensive with a heart rate of 115 beats per minute and a respiratory rate of 25 breaths per minute. The complete blood count revealed elevated white blood cells (15.130/μl) and an erythrocyte sedimentation rate of 105 mm. Serum electrolytes, hepatic and renal function tests were normal.

He reported that the symptoms had occurred after attending a party for teenagers, during which he had consumed a large quantity of alcohol and had an episode of vomiting. He also reported that he had practiced a fire-eating performance during the party using liquid paraffin, without knowledge of inhaling any of it. A chest radiograph showed infiltration in the right middle lobe which was diagnosed as aspiration pneumonia, and he was treated at the local health center with a combination of antibiotics (a macrolide and a second-generation cephalosporin). After 5 days of treatment, because of clinical deterioration, he was referred to a pulmonary clinic.

**Figure 1:**
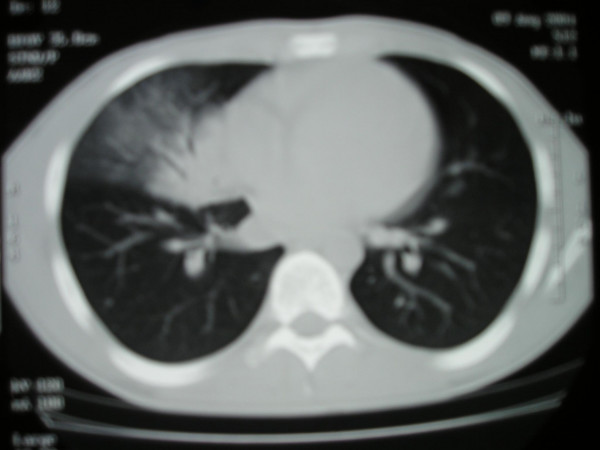
Computed tomography of the chest at admission showing consolidation with an air bronchogram in the right middle lobe and areas of atelectasis and ground glass opacities in the middle and lower right lobes.

Spirometry revealed severe restriction of lung function (a forced vital capacity 68% of the normal value). Arterial blood gas measurements were within normal ranges: pH = 7.38, pCO_2 _= 36.3 mmHg, pO_2 _= 98.5 mmHg and sO_2 _= 97.4%. A chest computed tomography scan was performed, which showed consolidation with an air bronchogram in the right middle lobe, and areas of atelectasis and ground glass opacities in the middle and lower right lobes (Figure 1).

**Figure 2:**
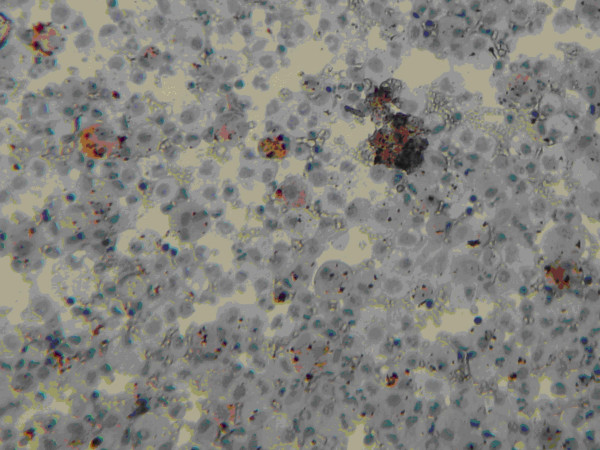
The bronchoalveolar lavage.

**Figure 3:**
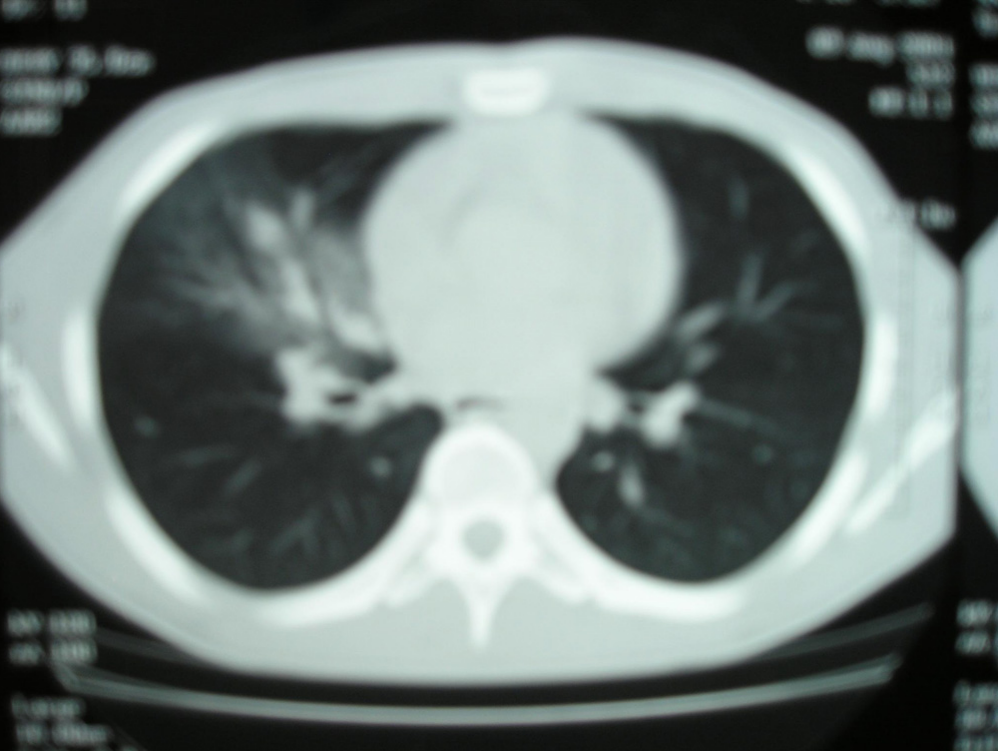
Computed tomography of the chest following 6 days of treatment showing significant improvement.

Bronchoscopy presented inflamed, hyperemic mucosa, especially on the right side. Bronchoalveolar lavage fluid was hemorrhagic and revealed cytoplasmic vacuolation of the macrophages, lipid-laden alveolar macrophages detected by lipid staining and oil-red-O stain, and neutrophilia (23%; Figure 2). Owing to the bronchoalveolar lavage fluid findings and the history of fire-eating, the patient was diagnosed with hydrocarbon pneumonitis and was treated with systemic steroids (intravenous prednisolone 25 mg × 2) and intravenous antibiotics. Steroids were prescribed for 21 days: 6 days during the patient's hospitalization followed by tapering doses over the next 15 days. There was significant clinical and radiologic resolution 6 days after treatment was initiated (Figure 3).

## Discussion

Aspiration of volatile hydrocarbons, also referred to as fire-eater's pneumonia, is an acute hydrocarbon pneumonitis occasionally seen in children and fire-eating performers. These hydrocarbons are members of the paraffin, naphthene and aromatic classes, and are characterized by low viscosity and surface tension. These substances have the ability to rapidly diffuse throughout the bronchial tree and disrupt the surfactant barrier. They then provoke the activation of macrophages, leading to an increased release of cytokines and a prolonged inflammatory reaction. Electron microscopic inspection of bronchoalveolar lavage fluids reveals a paucity of microorganisms, and macrophages with lipoid-containing inclusions that exhibit all morphologic signs of activation [2].

Hydrocarbon compounds with high viscosity and surface tension, such as mineral oil and petrolatum, have been implicated in a chronic lower respiratory tract illness called exogenous lipoid pneumonia, which is characterized by histologic findings that are similar to those of fire-eater's pneumonitis. Acute forms usually have a good outcome and regress favorably in a few days with conservative supportive measures; however, there have also been cases described of severe cavitary pneumonia and adult respiratory distress syndrome [3-6].

Fire-eater's pneumonia is generally a pseudo-infectious lung disease with an intense release of inflammatory cytokines; the use of steroids may improve the outcome in severely affected patients. However, this treatment is not standard and has been reported as not being reliably effective, thus the additional therapy of steroids in such a case remains controversial [1]. Some studies also suggest the use of gastric decontamination to prevent subsequent pulmonary injury from hydrocarbon ingestion.

## Conclusion

In conclusion, hydrocarbon pneumonitis should be included in the differential diagnosis of pneumonias. Typical clinical symptoms include dyspnea, cough, hemoptysis, chest pain and fever. Radiographic findings include unilateral or bilateral lung consolidation, well-defined nodules, pneumatoceles (well-defined cavitary nodules), pleural effusion, and spontaneous pneumothorax [7,8].

Recent exposure to volatile hydrocarbons provides a basis for clinical diagnosis, as symptoms and radiologic findings are not specific. Even small quantities of hydrocarbons can provoke a pneumonitis. As in the case reported here, the patient may not be aware of having aspirated liquid during the fire-eating performance, leading to an incorrect diagnosis and delaying the correct diagnosis.

## Competing interests

The authors declare that they have no competing interests.

## Consent

Written informed consent was obtained from the patient for publication of this case report and accompanying images. A copy of the written consent is available for review by the Editor-in-Chief of this journal.

## Authors' contributions

EM drafted the definitive version of this manuscript, VV helped to draft the manuscript, DA participated in data collection and treated the patient. DP examined the bronchoalveolar lavage fluid specimen, TK conducted the bronchoscopy, AS performed the histological photography and rendered an interpretation, EV evaluated the chest radiograph and computed tomography scan, PC conceived the study and participated in its design and coordination. All authors read and approved the final manuscript.
